# Identification and Characterization of *Verticillium longisporum* Lineage A1/D1 from *Brassica* Crops in Manitoba, Canada

**DOI:** 10.3390/ijms21103499

**Published:** 2020-05-15

**Authors:** Zhongwei Zou, Vikram Bisht, W. G. Dilantha Fernando

**Affiliations:** 1 Department of Plant Science, University of Manitoba, 66 Dafoe Road, Winnipeg, MB R3T 2N2, Canada; zhongwei.zou@umanitoba.ca; 2 Primary Agriculture Branch, Manitoba Agriculture, Carman, MB R0G 0J0, Canada; vikram.bisht@gov.mb.ca

**Keywords:** Brassica crops, Brassica napus, Raphanus sativus, Brassica juncea, Verticillium longisporum

## Abstract

Verticillium stripe in canola (*Brassica napus* L.) caused by *Verticillium longisporum* was first reported in Manitoba in 2014. In this study, *Brassica* crops including canola, mustard (*Brassica juncea*) and radish (*Raphanus sativus*) with visible symptoms of Verticillium stripe were collected from Portage La Prairie, Manitoba, and the pathogens were isolated. Isolates from canola and radish were identified to *V. longisporum*, which produced longer conidia (7.92–12.00 µm) than *Verticillium dahliae* (4.32–7.04 µm). An isolate derived from mustard was characterized as *V. dahliae*. Molecular diagnostics with 18S rDNA, 5.8S rDNA and mating-type marker primers were used to confirm the identification of *Verticillium* isolates. PCR-RFLP of the mitochondrial small subunit rDNA and the cytochrome *b* gene were also employed to distinguish *V. longisporum* isolates from *V. dahliae*. The multi-gene characterization approach allowed for lineage determination, and *V. longisporum* isolates from canola and radish were in the A1/D1 group. Isolates of *Verticillium longisporum* from canola inoculated onto the canola cultivar ‘Westar’ caused symptoms of stem striping, stunting and short plants. Re-isolated fungal strains from infected stems were again inoculated onto canola plants, in order to confirm that *V. longisporum* was the causal agent of Verticillium stripe disease in the pathogenicity test.

## 1. Introduction

Canola (*Brassica napus* L. AACC, 2*n* = 38) is a relatively young species derived from a spontaneous interspecific hybridization between *Brassica rapa* L. (AA, 2n = 20) and *Brassica oleracea* L. (CC, 2*n* = 18) [1]. Canola is one of the most important oilseed crops worldwide, especially in Canada, where approximately 21.3 million tonnes were produced in 2017 (Canola Council of Canada 2018). Mustard (*Brassica juncea* L. AABB, 2*n* = 36) is an amphidiploid derived from the combination of two diploid genomes (*B. rapa* and *B. nigra* L. BB, 2*n* = 16). Radish (*Raphanus sativus* L. 2*n* = 2*x* = 18) is a member of the family Brassicaceae, and is closely related to *B. rapa* [2]. Verticillium stripe in canola, which is caused by *Verticillium longisporum*, was first observed and confirmed in Manitoba in 2014 (Canola Council of Canada). A survey conducted by the Canadian Food Inspection Agency and the Canola Council of Canada confirmed the presence of the pathogen in canola stubble from British Columbia, Alberta, Saskatchewan, Ontario and Quebec (http://www.inspection.gc.ca/plants/plant-pests-invasive-species/diseases/verticillium-stripe/eng/1420746212959/1420746213803). 

A Verticillium species supposedly representing *V. longisporum* (but not named so) was first described on Brussel sprouts (isolate 111) in the UK by Isaac in 1957 [3]. *Verticillium longisporum* (ex. *Verticillium dahliae* var. *longisporum* Stark*)* was first identified from horseradish in Germany in 1961 [4]. The *V. longisporum* represents a distinct group compared to *V. dahliae* [4,5], and the distinction of the taxonomic group was confirmed by molecular analysis [6]. Verticillium stripe disease caused by *V. longisporum* on oilseed rape (canola) has been reported in Europe, specifically in France, Germany, Russia and Sweden [7,8]. Ikeda et al. (2012) found that *V. longisporum* is the major pathogen on Chinese cabbage in Japan [9]. 

*Verticillium dahliae*, a close relative of *V. longisporum* which causes vascular wilt symptoms in canola, was first reported in North America in 2017 [10]. *Verticillium dahliae* has an extensive plant host range, and can cause Verticillium wilt disease in more than 200 economically important plant species, including potato (*Solanum tuberosum* L.), sunflower (*Helianthus annuus* L.) and canola [8,11,12,13], while *V. longisporum* is mostly restricted to oilseed rape in Europe [8]. *Verticillium longisporum* does affect other *Brassica* crops, including broccoli, cauliflower, cabbage, field mustard, horseradish, radish and turnip in Europe, the United States and Japan [14,15,16]. Verticillium stripe is a monocyclic and soil-borne fungal disease. This pathogen can enter the vascular system directly through the roots, or through hyphal penetration of an open wound in the roots. Hyphae and conidia are produced in the xylem, and move through the vascular system. Peeling off the outer skin of the stem on infected plants reveals blackening on the inside of the stem and multicellular microsclerotia (https://www.canolacouncil.org/canola-encyclopedia/diseases/verticillium/#fnote6, Canola Council of Canada). 

Molecular studies indicate that *V. longisporum* has an unusual evolutionary history. *Verticillium longisporum* is a diploid derived from the hybridization of at least three different lineages representing different parental species (*V. longisporum* A1/D1, A1/D2 and A1/D3). The three lineages are hybrids of four ancestral lineages, including the hitherto undescribed *Verticillium* species A1 and species D1, and two *V. dahliae* lineages referred to as *V. dahliae* D2 and D3. The resulting *V. longisporum* lineages are A1/D1, A1/D2 and A1/D3 [8]. Eynck et al. (2009) indicated that *V. longisporum* was more virulent on oilseed rape than *V. dahliae*, due to its hybrid ancestry [17]. Enhanced virulence may result from lineage-specific genetic and epigenetic changes of involved ancestors generated after hybridization [18]. The objectives of this study were to identify and characterize *Verticillium* isolates from *Brassica* crops, including canola, mustard and radish, by morphological observation and molecular analysis. The lineages of these characterized isolates were confirmed with a multiplex polymerase chain reaction (PCR) assay. We also performed greenhouse pathogenicity tests of a *V. longisporum* isolate to understand its potential threat and effects on canola. 

## 2. Results

### 2.1. Pathogen Isolation

Naturally infected sampled stems exhibited black striping on the stem with microsclerotia. Twenty-nine Verticillium isolates from canola, one isolate from radish and one isolate from mustard were obtained (Table S1). 

### 2.2. Morphological Identification

**Figure 1:**
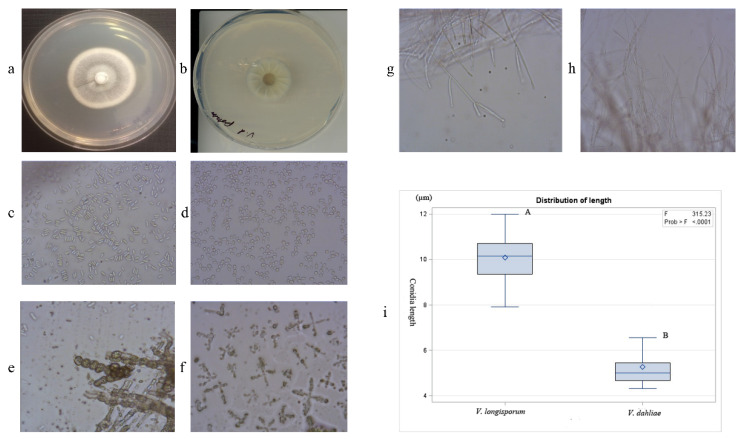
Morphological comparison of isolate VL-H1 of *Verticillium longisporum* (**a**,**c**,**e**,**g**) with Vd1396-9 of *Verticillium dahliae* (**b**,**d**,**f,h**). (**a,b**) Culture characteristics of *Verticillium* isolates; (**c**,**d**) Conidia observation (×40); (**e**,**f**) Microsclerotia (×40); and (**g**,**h**) Hyphae (×40). (**i**) Statistical analysis of *V. longisporum* and *V. dahliae* conidia length.

*Verticillium* isolate VL-H1 was obtained from canola tissue and grown on potato dextrose agar (PDA) for 14 days, which produced white colored mycelia on the top of cultures (Figure 1a). The *V. dahliae* isolate displayed greyish colonies with radiating ridges (Figure 1b). The mean length of *Verticillium* isolate VL-H1 conidia was significantly longer than that of the *V. dahliae* isolate (Vd1396-9) (Figure 1c,d,i). The *Verticillium* isolate from canola (VL-H1) produced longer conidia, ranging from 7.92 to 12.00 µm (mean 10.08 µm), while the mean length of *V. dahliae* isolate conidia was 5.27 µm and ranged from 4.32 to 7.04 µm (Figure 1i). Under the microscope, the *Verticillium* isolate VL-H1 from canola produced irregularly-shaped microsclerotia (Figure 1e), while colonies of the *V. dahliae* isolate (Vd1396-9) formed regularly-shaped microsclerotia (Figure 1f). Isolated VL-H1 produced more branched hyphae, while the *V. dahliae* isolate produced less profusely branched hyphae (Figure 1g,h). Taken together, the colony growth, conidia length, microsclerotia and hyphal traits suggest that the *Verticillium* isolate VL-H1 was *V. longisporum*. The other 28 isolates that were derived from canola and the one isolate identified from radish all exhibited similar morphological characteristics, suggesting that they too were *V. longisporum*. However, one isolate retrieved from mustard displayed morphological traits similar to *V. dahliae*. These results were further validated through molecular identification. 

### 2.3. PCR Characterisation

**Figure 2:**
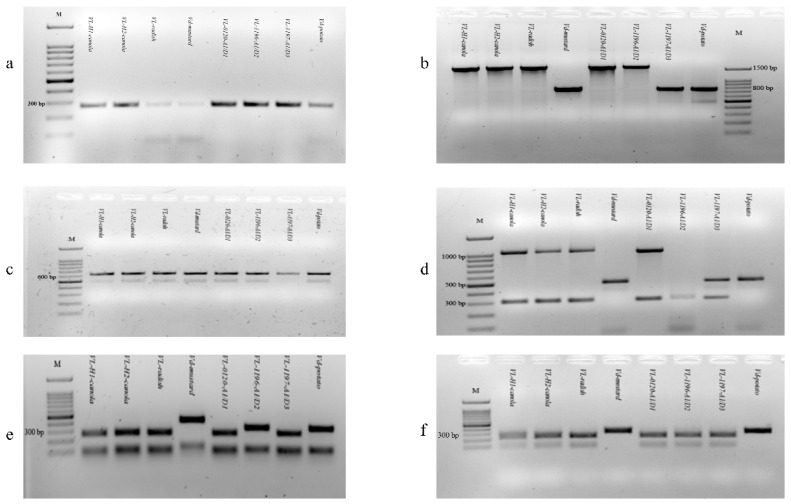
PCR amplification of 18S rDNA intron region (**a**); 18S rDNA (**b**); and mating type 1-1-1 (**c**) in *Verticillium longisporum* and *Verticillium dahliae* isolates. (**d**) Multiplex PCR amplification in *Verticillium longisporum*-*Verticilliun dahliae* isolates. (**e**,**f**) PCR-AFLP analysis of mitochondrial mtSSU-rDNA (**e**) and cytochrome b gene (**f**) digested with restriction enzymes MseI and Dde I, respectively. In all agarose gels: Lane 1, DNA ladder (100bp); Lanes 2–3, *V. longisporum* isolates from VL-H1-canola and VL-H2-canola (VL-H1 and VL-H2); Lane 4, *V. longisporum* isolate from radish (VL-R); Lane 5, *V. dahliae* isolate from mustard (Vd-M); Lanes 6–8, control isolates from different lineages of *V. longisporum* isolates including VL-0120-A1/D1 (PD638), VL-1196-A1/D2 (PD629), and VL-1197-A1/D3 (PD687); Lane 9, *V. dahliae* isolate from potato (Vd-potato (Vd1396-9)).

Polymerase chain reaction (PCR) amplification of the 18S rDNA intron region with the primers VlspF1/VlspR4 produced a 275 bp band in all the tested *V. longisporum* and *V. dahliae* isolates (Figure 2a). *Verticillium longisporum* isolates derived from canola and radish produced a 1598 bp band, while the *V. dahliae* isolates from mustard produced a 757 bp amplicon when amplified with the primers VeruniF2/VeruniR3, designed for the 18S rDNA region. However, the 18S rDNA region cannot be used to distinguish the *V. longisporum* isolate A1/D3 group from *V. dahliae* isolates, since both produced the same amplicon (757 bp) (Figure 2b). Nucleotide sequence analysis of 18S rDNA indicated that isolates derived from canola and radish had 99% similarity with *V. longisporum*, while the sequence identity of an isolate identified from mustard was similar to *V. dahliae* (data not shown). All isolates were categorized as mating type 1-1, since only the VdMAT1-1-1F01/VdMAT1-1-1-R01 primer designed for mating type1-1 was amplified (Figure 2c). 

*Verticillium* isolates derived from canola and radish produced 1020 bp ITS and 310 *EF -1α* gene amplicons, similar to the *V. longsiporum* control isolates from the A1/D1 group (Figure 2d). The *Verticillium* isolate from mustard produced a 490 bp *GPD* gene, similar to the *V. dahliae* isolate from potato. With this multiplex PCR assay, we were able to differentiate the *V. longisporum* isolate from the A1/D3 group, which produced both *GPD* and *EF -1α* genes with 490 bp and 310 bp bands, from the *V. dahliae* isolate, which only produced the 490 bp *GPD* gene (Figure 2d). The *V. longisporum* isolates from the A1/D2 group yielded only a 310 bp amplicon (*EF -1α* gene) (Figure 2d). All other *Verticillium* isolates from canola were characterized as the *V. longisporum* A1/D1 group. 

The *Verticillium* isolates from canola and radish, as well as the *V. longisporum* isolates from the A1/D1 or A1/D3 groups, produced a 258 bp mtSSU-rDNA band, and the *V. dahliae* isolate from potato and *V. longisporum* isolate from the A1/D2 group produced 317 bp bands (Figure 2e). However, the *V. dahliae* isolate from mustard produced a 413 bp band, which was different from the *V. dahliae* potato isolate (Figure 2e). Furthermore, for the cytochrome *b* gene, all *V. longisporum* isolates produced a 226 bp band, whereas *V. dahliae* isolates from mustard and potato both produced a 320 bp band (Figure 2f). 

### 2.4. Disease Assessment 

**Figure 3:**
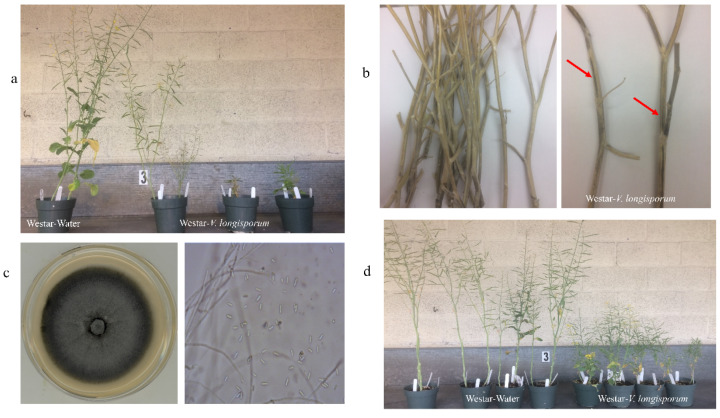
Pathogenicity test of *Verticillium longisporum* isolate (VL-H1) on canola susceptible cultivar ‘Westar’. Westar plants inoculated with water were included as controls. (**a**) Westar plants inoculated with *V. longisporum* isolate show a shorter height, stunting, leaf yellowing and senescence. (**b**) After harvest, the infected stems show black striping and microsclerotia. (**c**) Example of one *V. longisporum* isolate recovered from an infected canola stem. Isolate was grown on PDA plate, and conidia of isolate was observed under microscope. (**d**) Disease symptoms of Westar plants inoculated with recovered *V. longisporum* isolates. Westar plants inoculated with water were set as a non-disease control.

The Westar plants inoculated with *V. longisporum* showed disease symptoms in the form of yellowing and chlorosis at 7 and 14 days post-inoculation (dpi) (Table 1). The mean scores of disease severity were significantly higher in the plants infected with the *V. longisporum* isolates identified in this study than in those inoculated with water after 14 and 21 dpi (Table 1). After 35 dpi, the plants inoculated with *V. longisporum* produced symptoms such as a short height, leaf yellowing, chlorosis, leaf senescence and plant stunting (Figure 3a, Table 1), and approximately 17% of the *V. longisporum*-inoculated plants were dead. The *V. longisporum* and *V. dahliae* isolates, identified from radish and mustard, respectively, caused similar symptoms and disease severity on canola Westar plants (Table 1). After harvest, the stems infected with *V. longisporum* displayed streaking, striping or blackening, and microsclerotia were observed on the stems (Figure 3b). *V. longisporum* isolates were recovered and confirmed from the infected stems of the canola plant, showing similar morphological characteristics (Figure 3c). Westar plants were inoculated by the recovered *V. longisporum* isolates, and showed dwarfed and stunted plant growth, which indicated that the recovered *V. longisporum* isolate is the causal agent of Verticillium stripe disease in canola (Figure 3d). 

## 3. Discussion

This study identified and characterized *Verticillium* isolates from *Brassica* crops in Manitoba, Canada. A total of 29 isolates derived from canola were identified as the *V. longisporum* A1/D1 group by morphological and molecular characterization. To the best of our knowledge, this is the first scientific peer-reviewed report on the *Verticillium* disease in canola and radish caused by *V. longisporum* in Canada. 

As described previously by Tran et al. (2013), *V. longisporum* A1/D1 strains have an intron within the 18S rDNA region, while *V. longisprum* A1/D3 strains have the same 18S rDNA and 5.8S rDNA as *V. dahliae* [19]. Therefore, we could not differentiate the *V. longisporum* A1/D3 isolates from *V. dahliae* using the rDNA region. All *V. longisporum* isolates (30) identified from canola and radish were characterized to the A1/D1 group and carried a *MAT1-1* idiomorph. The polymerase chain reaction (PCR) identification of *Verticillium* isolates indicated that the multiplex PCR was the best assay, since it not only characterized the different *V. longisproum* lineages, but also differentiated them from the *V. dahliae* isolate. *Verticillium longisporum* and *Verticillium albo-atrum* produced 258 bp mtSSU-rDNA PCR-RFLP, while a 317 bp band was detected in Japanese isolates of *V. dahliae* [16]. However, the *V. dahliae* isolate from mustard identified in this study gave a longer digested fragment than the isolate from potato, indicating diversity among *V. dahliae* isolates from different host plants. As shown in this study, the PCR-RFLP genotyping assay with mtSSU-rDNA cannot differentiate the *V. longisporum* A1/D2 group from *V. dahliae*. At the same time, the PCR-RFLP genotyping assay using the cytochrome *b* gene did successfully differentiate *V. longisporum* and *V. dahliae* isolates. Thus, the multiplex PCR assay is recommended for the successful molecular identification of *Verticillium* species. In addition, the morphological differences between *V. longisporum* and *V. dahliae*, e.g., significantly longer conidia, irregular microsclerotia and more branched hyphae in *V. longisporum*, can assist identification. 

*Verticillium longisporum* caused pale black stripes on the stem, as well as plant stunting, leaf senescence and microsclerotia formation close to harvest (Figure 3b). Pathogenicity testing using a *V. longisporum* A1/D1 isolate from canola indicated that inoculation with *V. longisporum* caused significantly greater disease severity after three weeks than the water control (Table 1). Since some of the disease symptoms scored were leaf yellowing or blackening veins, the canola plants experiencing regular leaf senescence were also rated as 2 or 3 in later stages of growth. This likely caused the scoring of the disease at 28 and 35 days post-inoculation (dpi) in canola plants inoculated with water. *Verticillium longisporum* A1/D1 is considered the most virulent group across Brassicaceae crops, especially on oilseed rape [20]. *Verticillium longisporum* A1/D1 was identified from cauliflower, eggplant and watermelon in California [20,21]. *Verticillium dahliae* has limited colonization capacity on canola, and can cause wilting at the seedling stage [10]. *Verticillium longisporum* is more virulent on Brassicaceae crops, which are its main hosts. *Verticillium dahliae* generally has a wider host range, and may affect other plant species more severely [8,11]. To the best of our knowledge, this is the first report to show *V. longisporum* lineage A1/D1 as a pathogen on oilseed rape in a North American field. *Verticillium longisporum* lineage A1/D2, which is considered the least virulent lineage of *V. longisproum*, was reported on horseradish in Illinois, in close proximity to the main oilseed rape-growing regions of the United States (Minnesota, North Dakota and Oklahoma) [20]. Canola acreage in the prairies had topped 22 million acres by 2017 (https://www.canolacouncil.org/markets-stats/statistics/harvest-acreage/). Identification of *V. longisporum* lineage A1/D1 isolates from canola and radish in Canada highlight the potential risk of Verticillium stripe disease in canola fields in the future as a threat to the trade of oil, seed and meal to global markets, especially considering that *V. longisporum* microsclerotia can survive in soil for up to 15 years, and has a wide range of Brassicaceae host species (https://www.canolacouncil.org/canola-encyclopedia/diseases/verticillium/#fnote6, Canola Council of Canada). Our study indicates that *V. longisporum* can cause significant yield losses and reduce oil quality. Ancestral species harboring A1 and D1 have not yet been identified, and they may come from non-agricultural hosts or non-pathogenic saprotrophs [8,14]. Since this is the first study to report *V. longisporum* lineage A1/D1 in canola and radish fields in Canada, surveys of this disease in fields of other Brassicaceae species are warranted. A recent study indicated that the ‘*V. longisporum* A1/D1 West’ population is genetically more diverse than the ‘A1/D1 East’ isolates, based on their geographic origins in Europe, which caused the sudden emergence of Verticillium stripe disease in the UK [22]. Understanding the genetic and population diversity of *V. longisporum* isolates derived from Brassica crops from east to west North America will help to identify the origins, the genetic basis of the hybridization process and the host range of *V. longisporum* lineage A1/D1. 

Currently, no effective fungicides are registered to control Verticillium stripe disease in canola. The identified *V. longisporum* isolates will be used for *B. napus* resistance screening in both breeding varieties and commercial cultivars. Introducing resistance from *Brassica carinata* or *Brassica oleracea* (low susceptibility) is also an option for *B. napus* breeding programs to overcome Verticillium stripe disease [23,24]. Proper crop rotation, biosecurity practices, new fungicides, weed management, increasing soil fertility and resistance breeding will help to develop a successful control strategy against this soil-borne disease. 

## 4. Materials and Methods 

### 4.1. Sample Collection and Fungal Pathogen Isolation

Stems of *Brassica* crops such as canola, mustard and radish showing *Verticillium* disease symptoms were collected in Portage La Prairie, Manitoba in 2016. The striped stems were peeled and cut into small pieces (1 cm long). The small diseased pieces were surface-sterilized by dipping into a bleach solution of 10% NaOCl with 20 µL Tween-20 (Sigma, Toronto, ON, Canada) for 2 min. The sterilized sections were then rinsed in sterile distilled water for 2 min, soaked in 70% ethanol for 2 min and washed again with sterile distilled water for another 2 min. The stem sections were placed in Petri dishes containing potato dextrose agar (PDA) and incubated at room temperature. Fungal colonies growing out of the diseased tissues were purified using the single-spore technique, and cultured on PDA for two weeks. An isolate of *V. dahliae* from potato (Vd1396-9) was obtained from Dr. Fouad Daayf (Plant Science, University of Manitoba), and included as a reference isolate for this study. To categorize the lineages of *V. longisporum*, three reference isolates were included, one representing each of the three species A1/D1, A1/D2 and A1/D3. Reference lineages were kindly supplied to us by Drs. A. Tiedemann (Department of Crop Science, University of Gottingen) and K. Subbarao (Department of Plant Pathology, University of California, Davis) (Table S1). 

### 4.2. Morphological Identification of Isolates

A representative isolate of *V. longisporum* from canola, VL-H1, which was obtained in this study, and a reference isolate of *V. dahliae* from potato (Vd1396-9) were grown and maintained on PDA at room temperature for two weeks. The colony morphology, conidia shape, microsclerotia and hyphae of isolates were observed under a microscope (Fisher Science Education), and used to differentiate the *Verticillium* species [5,6]. Each of the 30 conidia from 14-day-old cultures of *V. longisporum* and *V. dahliae*, respectively were measured under the microscope, using a micrometer to determine conidia length. 

### 4.3. DNA extraction, PCR Amplification and PCR-Restriction Fragment Length Polymorphism

Genomic DNA from 29 *Verticillium* isolates obtained from canola, radish and mustard, and reference isolates (Table S1) were extracted from a mixture of mycelia, microsclerotia and conidia suspensions using a modified CTAB method [25]. The extraction buffer was briefly aliquoted to the harvested mixture and homogenized. After incubation at 65 °C for 30 min, DNA was extracted with phenol/chloroform/isoamyl (25:24:1). The supernatant was then precipitated and washed with 95% and 70% ethanol, respectively. Finally, the DNA pellet was dissolved in 100 µL DNase-free water.

To characterize the species of *Verticillium* isolates, the primer sets VlspF1/VlspR4 and VerniF2/VernR3 were used for amplifying the 18S rDNA intron and 18S rDNA, respectively [9,15]. In addition, two primer sets of VdMAT1-1-1F01/VdMAT1-1-1-R01 and VdMAT1-2-1F02-2/VdMAT1-2-1-R02-2 were used for *Verticillium* spp. mating type gene amplification [16]. All primers used are listed in Table 2. The polymerase chain reaction (PCR) amplification was conducted in a total volume of 25 µL, with a reaction mixture containing 25 pmol of each primer, 3.75 mM MgCl_2_, 1.25 mM of each deoxynucleoside triphosphate (dNTPs) and 1.25 U EconoTaq DNA polymerase (Econo Taq DNA polymerase, Lucigen, Middleton, WI) with 2.5 µL supplied 10 × PCR reaction buffer, and a 10 to 50 ng DNA template. The PCR thermocycling began with a 3-min first step at 95 °C for denaturation, and then 35 cycles of amplification at 95 °C for 30 s, annealing temperature for 30 s and extension at 72 °C for 1 min, and then completed with a final extension step at 72 °C for 5 min. The PCR products were separated on a 2.0% agarose gel stained with Redsafe (iNtRON BIOTECHNOLOGY, Seongnam-Si, Korea). 

A multiplex PCR assay developed by Inderbitzin et al. (2013) was used to identify the lineage of the characterized *Verticillium* isolates [26]. Briefly, the primer mixture was prepared by adding 6.25 µL each of D1f/AlfD1r (*GPD* gene, *glyceraldehyde-3-phosphate dehydrogenase*), 6.25 µL each of A1f/A1r (*elongation factor 1-alpha*) and 3.25 µL each of Df/Dr (ITS, internal transcribed spacer region) (Table 2), and then finally adding 93.75 µL sterile water to a total volume of 125 µL. The initial concentration of mixed primers was 100 µM. The PCR reaction was conducted in a 25 µL volume, which included 2.5 µL of mixed primers, 2.5 mM dNTPs, 2.5 µL of supplied 10 × PCR reaction buffer, 2U EconoTaq DNA polymerase (Econo Taq DNA polymerase, Lucigen, Middleton, WI, USA) and a 50 ng DNA template. The PCR program comprised a 2-min initial denaturation at 94 °C; 35 cycles of amplification at 94 °C for 10 s, 54 °C for 20 s and 72 °C for 1 min, and a final extension at 72 °C for 7 min. 

To further confirm the identification of *Verticillium* isolates, regions of the mitochondrial small-subunit rRNA gene and the cytochrome *b* gene were amplified with the primer pairs NMS1/NMS2 and cobF1/cobR1, respectively [9,27] (Table 2). The amplicons of 584 bp for mtSSU-rDNA and 320 bp for the cytochrome *b* gene were then digested using the restriction enzymes MseI and DdeI (New England BioLabs, Ipswich, MA, USA), respectively. The digested products were separately visualized in 1.5% agarose gels, as described above. 

### 4.4. Pathogenicity Tests of V. longisporum on Canola

Pathogenicity was tested on *B. napus* cultivar Westar, a spring canola cultivar which is mostly susceptible to fungal pathogens in Canada. To prepare the inoculum, identified Verticillum isolates were grown on a PDA medium for 10 days at room temperature. Five agar discs were transferred to 250 mL flasks of sterile potato dextrose broth, and incubated for two weeks at room temperature with shaking (120 rpm) in the dark. The conidia suspension was harvested by filtering with sterilized Miracloth (Millipore Sigma, Oakville, ON, Canada), and the concentration was adjusted to 1 × 10^6^ conidia mL^−1^ with sterile distilled water on the day of inoculation. Four-week-old seedlings of cv. Westar were gently uprooted and washed with tap water to clean soil off the roots. The roots of 16 seedlings were then immersed in 250 mL of the conidia suspension for 30 min [16,20], and then transplanted to pots. Controls for the pathogenicity test included 16 seedlings that were dipped in water for 30 min and then transplanted to pots. After treatments, all the plants were placed in a growth chamber at 16 °C (night) and 21 °C (day), with a 16 h photoperiod. The experiment was repeated once in the greenhouse to check data reliability. After maturing, the infected stems of canola were subjected to the same method to recover the isolates from infection sites, as described above. Then, the recovered isolate was inoculated onto the Westar plant again to confirm that the *V. longisporum* isolate was a causal agent of the disease.

Plant disease severity was scored weekly for 35 days post-inoculation (dpi) using a key based on a 1–9 scale: 1 = no disease symptom; 2 = symptoms of yellowing on lower, older leaves; 3 = slight yellowing on the next youngest leaves; 4 = about 50% of leaves showing symptoms; 5 = > 50% of leaves showing symptoms; 6 = up to 50% of leaves dead; 7 = > 50% of leaves dead; 8 = only apical meristem alive; and 9 = plant dead [28,29]. 

### 4.5. Statistical Analysis 

Statistical analysis of the conidia length of *Verticillium* isolates, the disease score of pathogenicity test, the *F* measurement of the distance between two repeats and canola phenotypes, including plant height, oil content, seed weight and glucosinolate contents, were conducted using SAS version 9.4 (SAS Institute, Inc., Cary, NC, USA).

## Figures and Tables

**Table 1:** Mean disease scores on *B. napus* susceptible cv. ‘Westar’ plants inoculated with *Verticillium* isolates compared to ‘Westar’ inoculated with water.

Inoculum.	Days Post-Inoculation (dpi)				
	7	14	21	28	35
Westar-water	1.00(±0.00) ^1^a ^2^	1.00(±0.00)a	1.00(±0.00)a	1.85(±0.00)a	2.15(±0.00)a
Westar-VL-H1	1.10(±0.00)a	2.05(±0.00)a	3.65(±0.00)b	4.85(±0.25)b	5.95(±0.21)b
Westar-VL-H2	1.00(±0.00)a	2.25(±0.00)a	2.95(±0.00)b	4.75(±0.22)b	6.15(±0.23)b
Westar-VL-H3	1.25(±0.00)a	2.15(±0.00)a	3.25(±0.10)b	4.20(±0.20)b	5.25(±0.18)b
Westar-VL-H4	1.15(±0.00)a	2.00(±0.00)a	3.75(±0.15)b	4.45(±0.17)b	5.75(±0.23)b
Westar-VL-H5	1.05(±0.00)a	2.25(±0.10)b	4.05(±0.20)b	4.95(±0.21)b	5.75(±0.24)b
Westar-VL-H6	1.00(±0.00)a	2.35(±0.12)b	3.85(±0.10)b	4.75(±0.22)b	6.05(±0.18)b
Westar-VL-H7	1.10(±0.00)a	2.00(±0.00)a	3.45(±0.00)b	4.95(±0.21)b	5.75(±0.20)b
Westar-VL-H8	1.15(±0.00)a	2.05(±0.10)a	3.35(±0.00)b	4.50(±0.15)b	5.75(±0.20)b
Westar-VL-H9	1.25(±0.00)a	2.00(±0.00)a	3.65(±0.10)b	4.70(±0.21)b	5.65(±0.16)b
Westar-VL-H10	1.20(±0.00)a	2.15(±0.10)a	3.75(±0.12)b	4.95(±0.24)b	5.85(±0.18)b
Westar-VL-H11	1.10(±0.00)a	3.05(±0.10)b	3.95(±0.12)b	4.65(±0.20)b	5.25(±0.15)b
Westar-VL-H12	1.05(±0.00)a	2.15(±0.09)a	3.75(±0.15)b	4.80(±0.22)b	5.75(±0.18)b
Westar-VL-H13	1.25(±0.00)a	2.25(±0.10)a	3.95(±0.10)b	5.15(±0.20)b	6.15(±0.18)b
Westar-VL-H14	1.00(±0.00)a	2.00(±0.00)a	3.45(±0.10)b	4.75(±0.19)b	5.75(±0.20)b
Westar-VL-H15	1.15(±0.00)a	2.25(±0.00)a	3.70(±0.10)b	4.95(±0.20)b	5.90(±0.20)b
Westar-VL-H16	1.00(±0.00)a	1.95(±0.00)a	3.45(±0.10)b	4.50(±0.15)b	5.65(±0.15)b
Westar-VL-H17	1.10(±0.00)a	2.25(±0.00)a	3.50(±0.10)b	4.75(±0.21)b	6.05(±0.22)b
Westar-VL-H18	1.20(±0.00)a	2.00(±0.00)a	3.50(±0.00)b	4.90(±0.25)b	5.85(±0.20)b
Westar-VL-H19	1.00(±0.00)a	2.15(±0.08)a	3.45(±0.20)b	4.75(±0.24)b	5.75(±0.20)b
Westar-VL-H20	1.20(±0.00)a	2.25(±0.00)a	3.75(±0.10)b	5.05(±0.22)b	5.45(±0.15)b
Westar-VL-H21	1.05(±0.00)a	2.45(±0.00)b	3.80(±0.20)b	4.95(±0.20)b	5.70(±0.23)b
Westar-VL-H22	1.20(±0.00)a	2.00(±0.00)a	3.50(±0.15)b	4.85(±0.25)b	5.85(±0.21)b
Westar-VL-H23	1.00(±0.00)a	2.05(±0.00)a	3.55(±0.11)b	4.45(±0.20)b	5.40(±0.15)b
Westar-VL-H24	1.50(±0.00)a	2.55(±0.05)b	3.75(±0.10)b	4.95(±0.19)b	6.15(±0.25)b
Westar-VL-H25	1.15(±0.00)a	2.25(±0.00)a	3.55(±0.10)b	4.55(±0.15)b	5.65(±0.20)b
Westar-VL-H26	1.15(±0.00)a	2.15(±0.00)a	3.50(±0.10)b	4.95(±0.22)b	5.80(±0.16)b
Westar-VL-H27	1.20(±0.00)a	2.05(±0.00)a	3.75(±0.10)b	4.75(±0.25)b	5.40(±0.25)b
Westar-VL-H28	1.00(±0.00)a	2.00(±0.00)a	3.25(±0.10)b	4.50(±0.20)b	5.05(±0.15)b
Westar-VL-H29	1.20(±0.00)a	2.15(±0.00)a	3.45(±0.00)b	4.80(±0.25)b	5.25(±0.11)b
Westar-VL-R ^3^	1.25(±0.00)a	2.45(±0.00)a	3.80(±0.11)b	5.05(±0.21)b	6.45(±0.25)b
Westar-Vd-M ^4^	1.00(±0.00)a	2.00(±0.00)a	3.25(±0.10)b	4.50(±0.21)b	5.25(±0.14)b

^1^ Standard error in brackets. ^2^ Values followed by different letters indicate significant differences at *p* ≤ 0.05 (LSD) for disease scores. ^3^ *Verticillium longisporum* isolate identified from radish. ^4^ *Verticillium dahliae* isolate identified from mustard.

**Table 2:** Primers used in PCR assays to confirm the identity of *Verticillium* species in this study.

Primer.	Sequence	Amplicon/Locus	Reference
VlspF1	AGCCTGAGTCACGAGAGATATGGG	18S intron	Banno et al. 2011 [15]
VlspR4	CAAACCACGCCACTGCATTCTCGT		
VeruniF2	TCGTAGTAGAAGCTCGGCCTCCGGTC	18S rDNA	Ikeda et al. 2012 [9]
VeruniR3	TAAGAAGTCGGCGTACTACCGGGGT		
VdMAT1-1-1F01	CAACTCCTGGACTCTCATCG	MAT1-1-1	Banno et al. 2015 [16]
VdMAT1-1-1R01	AAATGGTCTGAGTCGGGGGT		
VdMAT1-2-1F02-2	CTAAGGCTCCAAGTCAATCC	MAT1-2-1	Banno et al. 2015 [16]
VdMAT1-2-1R02-2	TGCTGCCACTTGTTCAAACG		
D1f	CCCCGGCCTTGGTCTGAT	*GPD gene* ^1^	Inderbitzin et al. 2013 [26]
AlfD1r	TGCCGGCATCGACCTTGG		
A1f	AAGTGGAGCCCCGTATCTTGAAT	*EF-1α* ^2^	Inderbitzin et al. 2013 [26]
A1r	CAACTGGCAACAGGGCTTGAAT		
Df	CCGGTCCATCAGTCTCTCTG	ITS	Inderbitzin et al. 2013 [26]
Dr	CTGTTGCCGCTTCACTCG		
NMS1	CAGCAGTGAGGAATATTGGTCAATG	mtSSU-rDNA	Li et al. 1994 [27]
NMS2	GCGGATCATCGAATTAAATAACAT		
cobF1	GTTGAATTTATCTGAGGAGG	Cytochrome *b*	Ikeda et al. 2012 [9]
cobR1	GGAGGAGTTTGCATTGGATTAGCC		

^1^ GPD gene: glyceraldehyde-3-phosphate dehydrogenase; ^2^ EF-1α: elongation factor 1-alpha.

## Supplementary Materials

Supplementary materials can be found at https://www.mdpi.com/1422-0067/21/10/3499/s1. **Table S1**. *Verticillium* strains identified from *Brassica* crops and reference isolates were used in this study.
