# Supplementary material for: Identification and Characterization of Verticillium longisporum Lineage A1/D1 from Brassica Crops in Manitoba, Canada
Source: Int J Mol Sci. 2020 May 15;21(10):3499. doi: 10.3390/ijms21103499 (PMC7278989; doi:10.3390/ijms21103499)
Supplement: Supplementary file 1 [file ijms-21-03499-s001.pdf]

International Journal of
Molecular Sciences

MDPI

Table 1. *Verticillium* strains identified from *Brassica* crops and reference isolates used in this study.

Strain ID.	Host	Verticillium Species	Lineage	Strain origin
VL-H1[1,2,3]	Canola	*V. longisporum*	A1/D1	Canola field, Portage La Prairie, Manitoba
VL-H21[2,3]	Canola	*V. longisporum*	A1/D1	Canola field, Portage La Prairie, Manitoba
VL-H3[2]	Canola	*V. longisporum*	A1/D1	Canola field, Portage La Prairie, Manitoba
VL-H4[2]	Canola	*V. longisporum*	A1/D1	Canola field, Portage La Prairie, Manitoba
VL-H5[2]	Canola	*V. longisporum*	A1/D1	Canola field, Portage La Prairie, Manitoba
VL-H6[2]	Canola	*V. longisporum*	A1/D1	Canola field, Portage La Prairie, Manitoba
VL-H7[2]	Canola	*V. longisporum*	A1/D1	Canola field, Portage La Prairie, Manitoba
VL-H8[2]	Canola	*V. longisporum*	A1/D1	Canola field, Portage La Prairie, Manitoba
VL-H9[2]	Canola	*V. longisporum*	A1/D1	Canola field, Portage La Prairie, Manitoba
VL-H10[2]	Canola	*V. longisporum*	A1/D1	Canola field, Portage La Prairie, Manitoba
VL-H11[2]	Canola	*V. longisporum*	A1/D1	Canola field, Portage La Prairie, Manitoba
VL-H12[2]	Canola	*V. longisporum*	A1/D1	Canola field, Portage La Prairie, Manitoba
VL-H13[2]	Canola	*V. longisporum*	A1/D1	Canola field, Portage La Prairie, Manitoba
VL-H14[2]	Canola	*V. longisporum*	A1/D1	Canola field, Portage La Prairie, Manitoba
VL-H15[2]	Canola	*V. longisporum*	A1/D1	Canola field, Portage La Prairie, Manitoba
VL-H16[2]	Canola	*V. longisporum*	A1/D1	Canola field, Portage La Prairie, Manitoba
VL-H17[2]	Canola	*V. longisporum*	A1/D1	Canola field, Portage La Prairie, Manitoba
VL-H18[2]	Canola	*V. longisporum*	A1/D1	Canola field, Portage La Prairie, Manitoba
VL-H19[2]	Canola	*V. longisporum*	A1/D1	Canola field, Portage La Prairie, Manitoba
VL-H20[2]	Canola	*V. longisporum*	A1/D1	Canola field, Portage La Prairie, Manitoba
VL-H21[2]	Canola	*V. longisporum*	A1/D1	Canola field, Portage La Prairie, Manitoba
VL-H22[2]	Canola	*V. longisporum*	A1/D1	Canola field, Portage La Prairie, Manitoba
VL-H23[2]	Canola	*V. longisporum*	A1/D1	Canola field, Portage La Prairie, Manitoba
VL-H24[2]	Canola	*V. longisporum*	A1/D1	Canola field, Portage La Prairie, Manitoba
VL-H25[2]	Canola	*V. longisporum*	A1/D1	Canola field, Portage La Prairie, Manitoba
VL-H26[2]	Canola	*V. longisporum*	A1/D1	Canola field, Portage La Prairie, Manitoba
VL-H27[2]	Canola	*V. longisporum*	A1/D1	Canola field, Portage La Prairie, Manitoba
VL-H28[2]	Canola	*V. longisporum*	A1/D1	Canola field, Portage La Prairie, Manitoba
VL-H29[2]	Canola	*V. longisporum*	A1/D1	Canola field, Portage La Prairie, Manitoba
VL-R[2,3]	Radish	*V. longisporum*	A1/D1	Radish field, Portage La Prairie, Manitoba
Vd-M[2,3]	Mustard	*V. dahliae*	-	Mustard field, Portage La Prairie, Manitoba
PD638 (0120)[3,4]	Oilseed rape	*V. longisporum*	A1/D1	University of Gottingen, Tiedemann Lab
PD629 (1196)[3,4]	Horseradish	*V. longisporum*	A1/D2	UC, Davis, Subbarao Lab
PD687 (1197)[3,4]	Horseradish	*V. longisporum*	A1/D3	UC, Davis, Subbarao Lab
Vd-Potato (Vd1396-9)[3,4]	Potato	*V. dahliae*	-	University of Manitoba, Daayf Lab

[1] VL-H1 was used for morphological identification.

[2] Verticillium isolates were used for multiplex PCR identification and finally categorized as *V. longisporum* A1/D1 (data not shown). As well, these isolate were included in pathogenicity test.

[3] Verticillium isolates were used for different PCR assays validation and PCR-RFLP characterization.

[4] Verticillium isolates were used as reference isolates of *V. dahliae* and different lineages of *V. longisporium*.
